# Clinical and Breed Characteristics of Idiopathic Head Tremor Syndrome in 291 Dogs: A Retrospective Study

**DOI:** 10.1155/2015/165463

**Published:** 2015-04-30

**Authors:** Linda G. Shell, John Berezowski, Mark Rishniw, Belle M. Nibblett, Patrick Kelly

**Affiliations:** ^1^Department of Clinical Sciences, Ross University School of Veterinary Medicine (RUSVM), P.O. Box 334, Basseterre, Saint Kitts and Nevis; ^2^Department of Biomedical Sciences, Ross University School of Veterinary Medicine (RUSVM), P.O. Box 334, Basseterre, Saint Kitts and Nevis; ^3^Veterinary Public Health Institute, Swiss Veterinary Faculty, University of Bern, Hochschulstrasse 4, 3012 Bern, Switzerland; ^4^Department of Clinical Studies, Cornell University, 602 Tower Road, Ithaca, NY 14853, USA

## Abstract

*Objective*. To establish signalment and phenomenology of canine idiopathic head tremor syndrome (IHTS), an episodic head movement disorder of undetermined pathogenesis. *Design*. Retrospective case series. *Animals*. 291 dogs with IHTS diagnosed between 1999 and 2013. *Procedures*. Clinical information was obtained from an online community of veterinary information aggregation and exchange (Veterinary Information Network, 777 W Covell Boulevard, Davis, CA 95616) and conducted with their approval. Information on breed, sex, age of onset, tremor description, mentation during the event, effect of distractions and drugs, diagnostics, presence of other problems, and outcome was analyzed. *Results*. IHTS was found in 24 pure breeds. Bulldogs, Labrador Retrievers, Boxers, and Doberman Pinschers comprised 69%; mixed breeds comprised 17%. Average onset age was 29 months (range: 3 months to 12 years). First episode occurred before 48 months of age in 88%. Vertical (35%), horizontal (50%), and rotational (15%) movements were documented. Possible trigger events were found in 21%. Mentation was normal in 93%. Distractions abated the tremor in 87%. Most dogs did not respond to antiepileptic drugs. *Conclusions and Clinical Relevance*. This retrospective study documents IHTS in many breeds including Labrador Retrievers, Boxers, and mixed breeds.

## 1. Introduction

Canine idiopathic head tremor syndrome (IHTS), also sometimes referred to as episodic rapid repetitive myoclonus, is generally regarded as a benign condition manifesting as episodic uncontrolled head tremors that start and stop spontaneously. These head tremors have been reported to occur in “vertical” (“yes”) or “horizontal” (“no”) directions. Affected dogs appear alert during the tremors [1]. The diagnosis has been based on signalment, history, and characteristic head tremors as described above. Recent reports in the literature have characterized IHTS phenomenology in Doberman Pinschers [2] and English Bulldogs [3]. Although the cause of IHTS is unknown, affected Doberman Pinschers were traced to a common sire suggesting the condition may be inherited [1].

The veterinary literature, including neurology textbooks, does not contain significant information about IHTS for the practicing veterinarian. Affected dogs may initially be considered to have seizure activity, since this is the closest condition that might explain the signs. Thus, treatment with antiepileptic drugs (AEDs) may be attempted. Noting numerous cases of IHTS described by members of an online veterinary database (Veterinary Information Network, 777 W Covell Boulevard, Davis, CA 95616; this study was conducted with approval from Veterinary Information Network and its members), we sought to gain more information about IHTS by analyzing data from these cases. Specifically, we sought to document affected breeds, detail the character (duration and frequency) and direction of the head movement, investigate effects of stressful events and treatments on IHTS episodes, and determine whether or not the life span of affected dogs was affected.

## 2. Materials and Methods

Data on IHTS cases was obtained in two surveys.

### 2.1. Survey 1

Clinical information was reviewed from veterinarians who participate in an online community of veterinary information aggregation and exchange (Veterinary Information Network, 777 W Covell Boulevard, Davis, CA 95616: this study was conducted with approval from Veterinary Information Network and its members). Archived cases were searched for by key words: head tremor syndrome, head bob, head bobble, head bobbing, head bobbers, bobble head, bobble doll, shakes head yes, and shakes head no; these are all descriptors that investigators (LGS and MR) had observed veterinarians use to describe clinical signs of IHTS. Cases with at least one key word were evaluated by two investigators (LGS and BMN or PK) to confirm that the following inclusion criteria were met: characteristic description of head tremors associated with IHTS (up and down motions or side to side motions), absence of limb or truncal tremors and other signs of cerebellar origin, short duration (seconds to minutes) of head tremors, intermittent occurrence of head tremors, and lack of other neurological signs. Inclusion was further supported by the opinion of one or more veterinary neurologists who agreed that the description of signs suggested a diagnosis of IHTS.

From the written data and from the available videos of cases, we created a database containing details of age, breed, sex, age of onset, tremor description and direction, duration (in seconds/minutes), frequency and timing of events, mentation during the event, effect of distraction and types of distractors, and diagnostics performed.

### 2.2. Survey 2

An online survey of veterinarians identified additional cases of IHTS from which data was collected and reviewed (LGS and MR). In the survey, veterinarians were given a written description of IHTS, viewed a video of IHTS in a dog, and were asked to provide information (age of onset of tremor, breed, sex, tremor description including frequency, direction, etc.) from their medical charts if they had managed a case that clinically fit the description of IHTS. In addition to the information collected in survey one, additional information was obtained on body position during tremors (standing, sternal, and lateral), presence of behavioral problems, other diseases and seizures, history of littermates with signs, drugs administered prior to or during the tremor, and outcome.

### 2.3. Statistical Analysis

Wilcoxon Rank Sum Tests, Fischer's Exact Tests, and Chi Squared tests were used to compare groups. All analyses were performed using standard statistical software (R: a language and environment for statistical computing: Version i386.3.1.0. R Core Team (2014); R Foundation for Statistical Computing, Vienna, Austria; URL http://www.R-project.org/). A value of *P* < 0.05 was considered significant.

## 3. Results

### 3.1. Study Population

Of 197 potential cases of IHTS reported in the online database between January 1999 and April 2013, descriptive information and data were sufficient to meet the inclusion criteria for 137 cases and of these, 50 had accompanying videos which were reviewed and confirmed to be representative of IHTS. Of the 174 responses from the online survey, 154 responses also met the inclusion criteria and were included in the study. Data obtained in the two surveys were very similar and hence were combined for the analyses reported below (291 cases).

### 3.2. Breed Data

Table 1 presents the breed data. Most dogs with IHTS were purebreds (84%; 243/291) with Bulldogs being the most commonly reported (37%, 107/291) followed by mixed breeds (16%, 48/291), Boxers (13%, 37/291), Labrador Retrievers (11%, 33/291), and Doberman Pinschers (8%, 24/291). Videos 1, 2, and 3 show the head tremors in a miniature Schnauzer, a Labrador Retriever, and a Boxer, respectively.

### 3.3. Age of Onset

Age of onset was available for 280 cases and varied from 3 to 144 months. Mean and median ages were 29 and 25 months, respectively. The majority (88%) of dogs had their first episode of IHTS before 48 months of age. Bulldogs had a statistically significantly earlier age of onset (average 24 months) compared to the averages for the other breeds (32 months) (*P* = 0.017).

### 3.4. Sex

Overall, fewer females (41%; 115/279) were documented with IHTS than males (59%; 164/279). The percent of females varied by breed from a low of 27% females among Doberman Pinschers to a high of 51% in Bulldogs (*P* = 0.158).

### 3.5. Description of Head Tremor

#### 3.5.1. Direction of Head Tremor

Horizontal head motions (“no”) occurred in half the dogs (115/229). Rotational motions were the least common, found only in 15% of the dogs (Table 2). There were no significant differences in the horizontal and vertical directions between the five most commonly affected breeds: Bulldogs, mixed breeds, Boxers, Labradors, and Dobermans (*P* = 0.306). Video 1 shows more of a rotational type of tremor in a miniature Schnauzer See Video 1 in Supplementary Materials available online at http://dx.doi.org/10.1155/2015/165463. Video 2 shows vertical head tremors in a Boxer and video 3 shows horizontal tremors in a Labrador Retriever.

#### 3.5.2. Mentation

Ninety-three percent (246/264) of cases were considered to be alert or responsive during the episode. Only a few veterinarians described their patients' mentation as confused or disoriented (12), anxious (3), or lethargic (3).

#### 3.5.3. Duration of Tremor

In the majority of affected dogs (82%; 202/245) the tremors lasted less than 5 minutes (Table 3) with only 15% (37/245) lasting between 5 minutes and 1 hour. Three dogs were reported to have tremors occurring continuously over 12–48 hours.

#### 3.5.4. Frequency of Occurrence

Frequency data is presented in Table 4. The most commonly reported occurrences were multiple times/day (26%; 65/246) and every few days (25%; 61/246). However 22% (55/246) of the cases occurred infrequently based on combined results for the categories of “sporadically”, “quarterly,” and “less frequently than 3-4 months.”

#### 3.5.5. Body Position and Relationship to Rest

Affected dogs could be found in standing (41 cases), sitting (34 cases), or sternal (40 cases) positions at the onset of the tremor. Tremors did occur in lateral recumbency (5) but less commonly. Additional unprompted information indicated that head tremors were observed while the dog was resting, sleeping or waking from sleep in 66 cases.

#### 3.5.6. Effect of Distractions

In 87% (142/163) of dogs, distractions caused the tremor to disappear for an undisclosed time. Distractors included voluntary turning of the head to one side or the other, calling the dog by its name, offering it food, making a sound so that the dog turned its head, and asking the dog to perform a task. More than one distractor was frequently reported as being helpful.

#### 3.5.7. Time of Day That Tremor Was Observed

Most veterinarians (76%) did not have information about the time of day the tremors occurred (220/291 cases). For 71 dogs, a time of day was known: 58% (41/71) had tremors occurring in the evening/night hours, 34% (24/71) in the daytime hours, and 8% (6/71) during both day and night hours.

### 3.6. Predisposing Factors

#### 3.6.1. Concurrent Medications

Forty-four percent (67/154) of affected dogs were receiving medications at the time of onset of IHTS. For the majority (85%; 57/67), these medications were heartworm and flea control products; the rest of medications were prednisolone, diphenhydramine, tramadol, antibiotics, fludrocortisone, oral cyclosporine, and various eye medications.

#### 3.6.2. Concurrent Illness, Trauma, or Disease

Concurrent illness, trauma, or stressful events were noted in 21% of patients (33/154). Traumatic events (hit by car, dog fight), surgeries (castration, skin tumor removal, and cherry eye repair), and medical conditions (allergies; upper or lower respiratory infection; gastrointestinal, skin, or urinary tract infection; heart failure; demodicosis; heartworm disease; bee sting; papilloma; and immune mediated hemolytic anemia) had been diagnosed within a week (18 cases) or a month (6 cases) of the IHTS onset. Nine cases experienced a stressful event such as moving or a change in household members, travel, kennel confinement, or shock collar use. Specific triggers (gastrointestinal upset, shock collar training, and thunderstorm phobia) for repeated IHTS signs were identified in 3 cases.

#### 3.6.3. Concurrent Behavioral Issues

History of behavioral problems was positive in 13% (20/153) of cases either prior to (10 cases), concurrently with (3 cases), or after (7 cases) the onset of IHTS. Behavioral problems included fear or aggression (8), thunderstorm phobia (4), separation anxiety (3), and unspecified problems (5).

#### 3.6.4. Family History

Familial history was unknown for 89% (137/154) of cases, reported positive for IHTS in 4/154 (2.6%), and reported negative for IHTS in 13. Of the 4 cases with familial information, the known affected dog had littermates (2) or unspecified family members (2) with IHTS.

### 3.7. Outcome

#### 3.7.1. Treatment

Treatment was documented for 43/254 (17%) dogs. A variety of medications were used including bromide (5), phenobarbital (20), diazepam (12), and calcium carbonate (3). Less commonly used treatments included various antibiotics, corticosteroids, supplements (fish oil, antioxidants), clonazepam, and diphenhydramine. Veterinarians reported possible positive responses for oral maintenance phenobarbital (5/20), diazepam (3/12), calcium carbonate (1/3), and fish oil (1/1).

#### 3.7.2. Neurological Diagnostics

Results of cerebrospinal fluid analysis and advanced brain imaging were normal in 8 of 15 cases evaluated and unknown for 7 cases.

#### 3.7.3. Development of Other Neurological Disorders

Other neurological disorders did not develop in the majority (95%; 146/154) of dogs after the onset of IHTS. The neurological signs or disorders that developed in 8 dogs (5%) were diverse and were comprised of seizures (3) and one case of each of the following metronidazole-induced ataxia that resolved when medication was discontinued, degenerative myelopathy, idiopathic Horner's syndrome, cervical spondylopathy, and episodic collapse. Of the 3 cases that developed seizure activity, one developed it 9 years after IHTS was recognized; brain neoplasia was suspected but not proven. Another case developed seizures 2 months after onset of IHTS and subsequent results of spinal fluid analysis and brain magnetic resonance imaging studies were normal.

#### 3.7.4. Long Term Outcome

Of the 135 dogs for which long term data was available, 91% were still alive; the remaining 9% had died or been euthanized for reasons unrelated to IHTS. All or most of the clinical signs of IHTS had resolved in 67% (90/135) while 24% (33/135) continued to have intermittent clinical signs.

## 4. Discussion

Currently the diagnosis of IHTS is a clinical one based on signalment (breed disposition), history (no exposure to toxins, intermittent nature of signs, etc.), lack of concurrent neurological and physical signs that might explain the tremors, and presence of characteristic head tremors occurring sporadically and of short duration. No diagnostic tests have been identified to date that allow a more specific diagnosis of IHTS. Likewise, no other diseases have been described to produce the same signs as IHTS. Only a few IHTS cases have been documented to have advanced diagnostics (spinal fluid analysis, brain scans) and in those no abnormalities were reported [2, 3]. Therefore, the diagnosis of IHTS in this study was based on inclusion criteria that fit with IHTS as has been defined in other published studies [2, 3]. The authors acknowledge the inherent weakness of relying upon case descriptions and survey data but the clinical signs and historical data were sufficiently characteristic enough to hypothesize a diagnosis of IHTS and to eliminate cases that were not IHTS.

While previous studies centered on Bulldogs [2] and Doberman Pinschers [3], with an anecdotal report that Bulldog and Boxer breeds were commonly affected [1], we found IHTS in 24 pure breeds and in a substantial number of mixed breed dogs (17%). Although there are no data on relative breed prevalence in the US, it did seem that certain breeds appeared overrepresented, mainly, Bulldogs (37%), Boxers (13%), Labrador Retrievers (11%), and Doberman Pinschers (8%). Our scant familial information and the propensity for certain breeds to be affected suggest a possible hereditary basis to the condition which is consistent with the report that affected Doberman Pinschers could be traced to a common sire [1].

Most dogs in our study had onset of signs at a mature age with 88% occurring before 4 years of age. This onset agrees with prior reports that suggested onset age was usually between 6 months and 3 years [1–3]. However it is important to note that age of onset varied from as early as 3 months of age to as old as 12 years.

The previously described phenomenology of IHTS in Bulldogs [3] and Doberman Pinschers [2] is generally similar to our findings across 24 breeds and mixed breed dogs. Generally, IHTS presents as head tremors in a vertical, horizontal, or rotary direction, usually in mature dogs under 4 years of age. Episodes are spontaneous in onset and sporadic in number and occurrence. Duration is variable from seconds to several hours but most episodes do not last longer than 5 minutes. Tremors can occur while the dog is standing, resting, or sleeping. Most dogs are alert during an episode but some are anxious or lethargic. No drug therapy was identified that influenced the condition.

A number of new phenomenological findings emerged in our study. First, we documented the presence of a rotational head tremor in 15% of cases. This might be similar to the 9.2% of affected Dobermans that Wolf et al. [2] described with motions in both vertical and horizontal directions, which could be construed as rotational [2]. The significance of direction of head tremors is unknown. Second, getting the dog's attention so that it moved its head seemed to make the tremor disappear or cease momentarily in 87% of our cases. Most of the distractors reported in this study as well as those in Wolf et al.'s [2] study would result in mental stimulation or a change in neck position. Why the head tremor would cease with a change in neck position is unknown but warrants further investigation into one proposed theory that IHTS involves the stretch reflex mechanism [4]. Finally, although the data were limited, there was more of a trend toward evening or nighttime occurrence. However, this could be explained by the time of day that most owners are home and interacting with or observing their pet.

The relationship of the head tremor with body/neck posture, rest, the sleep/wake cycle, and neck movement needs further investigation. In the Bulldog study, IHTS episodes occurred predominately at rest [3]. Likewise, many of the Doberman Pinschers were reported to be reclining or dozing when the IHTS started [2]. Dogs in our study were reported to manifest IHTS while resting or being in stationary positions (sitting, standing, or being in sternal or lateral recumbency). The condition did not appear to be initiated by movement.

This study confirmed that the majority of cases do not have any other neurological or behavioral problems. However, stressful events or concurrent illness or trauma, occurring predominately within a week of the tremor onset, was noted in about one-fifth of our cases. In prior studies, stressful events were associated with IHTS in 46.7% of Dobermans and in 7% of Bulldogs [2, 3]. Thus underlying diseases and environmental stressors should be investigated in any dog presenting with signs of IHTS.

Outcome obtained from 135 cases indicated that the majority of affected dogs were still alive (91%) and that none had died or been euthanized because of IHTS. Thus IHTS is not a life-threatening disease. Furthermore the intermittent head tremors eventually disappeared in the majority of affected dogs (67%) although the survey did not gather data on the time it took for signs to resolve. One might assume that if underlying diseases and environmental stressors are addressed, clinical signs may resolve. Intermittent signs were reported, however, to occur in some dogs.

Focal seizure activity has been mentioned as a possible cause of IHTS [2] but currently there is little to no data to support that possibility. Other authors [4] have noted that AEDs do not appear effective in treating this disorder; that appeared to be the case for the few dogs that were medicated with maintenance AEDs in our study. We acknowledge, however, that, given the sporadic nature and short duration of clinical signs, it would be difficult to access AED efficacy; likewise, for the same reasons, it would be difficult to justify using AEDs in these patients unless more obvious generalized seizure activity was documented.

Unfortunately, tremors have many possible causes. In humans, tremors are the most common type of movement disorders [5–7] but tremors can also occur with stereotypies, tics, and psychogenic disorders [8, 9]. Tremors can also be a part of other movement disorders such as essential tremor syndrome, cervical dystonia [10], and paroxysmal dyskinesias [11]. However, tremors associated with these movement disorders are not paroxysmal, nor is the head a commonly affected area. Thus at the present time, IHTS does not appear to be similar to known movement disorders in humans.

In summary, our study shows that IHTS can be found in a wide range of dog breeds. The data we collected more clearly defines the clinical presentation and signs. IHTS is a benign condition that frequently resolves spontaneously and is nonresponsive to treatment with a wide range of conventional drugs. The condition is of concern to dog owners, however, and further studies appear warranted to establish its pathogenesis.

## Supplementary Material

These 3 video examples demonstrate the three directions of the head tremors. Video 1 shows a rotational type of head tremor in a 2 year old miniature Schnauzer. In this case, 8 episodes, lasting about 25 seconds each, occurred over a 2 month period. The 3 year old spayed female Boxer in video 2 demonstrates the vertical ("yes") head tremors. She had at least 6 episodes within a 3 week period with durations that varied from 30 seconds to 3 minutes. Many of them started when she was in lateral recumbency as noted in the video. In video 3, a young male Labrador has short duration of 'no' or horizontal head tremors.

## Figures and Tables

**Table 1:** Breed distribution of 291 dogs with IHTS.

Breed	Percentage (number)
Bulldog	37% (107)
Mixed	16% (48)
Boxer	13% (37)
Labrador Retriever	11% (33)
Doberman Pinscher	8% (24)
Staffordshire Terrier	3% (9)
Basset Hound	1% (3)
Boston Terrier	1% (3)
Golden Retriever	1% (3)
Greyhound	1% (3)
Parson Russell Terrier	1% (3)
Beagle	0.6% (2)
Great Dane	0.6% (2)
Miniature Schnauzer	0.6% (2)
Vizsla	0.6% (2)
Australian Shepherd	0.3% (1)
Border Collie	0.3% (1)
English Springer Spaniel	0.3% (1)
German Shorthaired Pointer	0.3% (1)
Papillon	0.3% (1)
Pembroke Welsh Corgi	0.3% (1)
Redbone Coonhound	0.3% (1)
Rhodesian Ridgeback	0.3% (1)
Shetland Sheepdog	0.3% (1)
Standard Poodle	0.3% (1)

Total	100% (291)

**Table 2:** Direction of head tremor based on breeds affected.

Breed	Horizontal	Vertical	Rotary	Total
Bulldog	41% (32)	42% (33)	17% (13)	78
Boxer	59% (17)	34% (10)	7% (2)	29
Labrador Retriever	44% (11)	44% (11)	12% (3)	25
Doberman Pinscher	48% (10)	33% (7)	19% (4)	21
Other pure breeds	49% (16)	33% (11)	18% (6)	33
Mixed	67% (29)	19% (8)	14% (6)	43

Total	50% (115)	35% (80)	15% (34)	229

**Table 3:** Duration of head tremors.

Time	Percent (number)	Cumulative %
0 to 0.5 min	21% (52)	21
0.5 to 1.0 min	20% (48)	41
1-2 min	18% (43)	58
2–5 min	24% (59)	82
5–60 min	15% (37)	98
1-2 hr	1% (3)	99
>2 hr	1% (3)	100

Total	100% (245)	

**Table 4:** Frequency of occurrence of head tremors.

Frequency category	Percent (number)
Continuously over 12–24 hours	1% (3)
Multiple times/day	26% (65)
Once a day	9% (21)
Every few days	25% (61)
Weekly	7% (18)
Monthly	9% (23)
Quarterly	4% (9)
Less frequently than every 3-4 months	10% (24)
Sporadically	9% (22)

Total	100% (246)
